# Age-related hearing loss: An updated and comprehensive review of the interventions 

**DOI:** 10.22038/IJBMS.2023.72863.15849

**Published:** 2024

**Authors:** Elham Tavanai, Vida Rahimi, Mohammad Ehsan Khalili, Somayeh Falahzadeh, Masoud Motasaddi Zarandy, Ghassem Mohammadkhani

**Affiliations:** 1 Department of Audiology, School of Rehabilitation, Tehran University of Medical Sciences, Tehran, Iran; 2 Department of Audiology, School of Rehabilitation, Isfahan University of Medical Sciences, Isfahan, Iran; 3 Otolaryngology Research Center, Amiralam Hospital, Tehran University of Medical Sciences, Tehran, Iran

**Keywords:** Aging, Auditory dysfunction, Presbycusis, Prevention, Treatment, Medical interventionst

## Abstract

Aging causes progressive degenerative changes in many organs, particularly the auditory system. Several attempts have been conducted to investigate preventive and therapeutic strategy/strategies for age-related auditory dysfunction, such as maintaining a healthy lifestyle through good nutrition, lower anxiety levels, and noise exposure, different pharmacological approaches, gene and cell therapy, and other strategies. However, it is not clear which approach is the best to slow down these dysfunctions because several different underlying mechanistic pathways are associated with presbycusis which eventually leads to different types of this disease. A combination of several methods is probably required, whereas the effectiveness for some people needs to be monitored. The effectiveness of treatments will not be the same for all; therefore, we may need to have a unique and personalized approach to the prevention and treatment of ARHL for each person. In addition, each method needs to specify what type of presbycusis can prevent or treat and provide complete information about the extent, duration of treatment, persistency of treatment, side effects, and whether the approach is for treatment or prevention or even both. This paper reviews the updated literature, which targets current interventions for age-related hearing loss.

## Introduction

The aging process is most commonly associated with age-related progressive degenerative changes in many body organs, especially in the auditory system, known as age-related hearing loss (ARHL), also referred to as presbycusis (1, 2).

ARHL is one of the most common chronic disorders in the elderly population (2, 3). Although the pathogenesis of ARHL is not yet well known, it has been demonstrated that many functional and structural pathological changes occur in the aging process of the peripheral and central auditory systems (4). These pathological changes cumulatively lead to the decline of auditory function like reduced hearing sensitivity and several difficulties in localization, speech understanding in noise, and information processing that affect an individual’s psychosocial situation (2, 4, 5). The changes in the peripheral system include the cochlear aging process often accompanied by degeneration of the stria vascularis, the hair cells and spiral ganglion neurons, and pathological changes in central auditory pathways (2, 4, 6). ARHL does not occur uniformly in all individuals. This is because many extrinsic (e.g., exposures to environmental ototoxic agents and noise) and intrinsic factors (e.g., genetic predisposition) could influence the course and severity of this disorder (4, 6). 

Currently, there is no specific, FDA-approved otoprotective agent for ARHL. As a first step of prevention, it was suggested that controlling the modifiable risk factors such as changing lifestyle toward a life with lower noise exposure, less stress, and healthy nutrition could be one of the most effective accepted ways to reduce the risk of ARHL (7). Regarding ARHL, some studies found a relationship between anxiety and hearing loss among older adults (8-11). In addition, different studies have investigated the relationship between nutrition and ARHL. A recent review article also has examined the relationship between nutrition and ARHL and found that a healthy diet consisting of more polyunsaturated fatty acids and less saturated fat and regular use of fruits and vegetables containing many anti-oxidants can prevent ARHL (12). Similarly, a recent population-based study found that pro-inflammatory foods with high sugar, calorie content, and alcohol were found to be associated with a higher risk of ARHL. They suggest that changing lifestyle toward less pro-inflammatory foods is more effective than the protective effect of anti-inflammatory or anti-oxidant foods (13). A similar population-based study also showed that higher carbohydrate and sugar intakes were associated with incident hearing loss in older adults(14). It can be clearly stated that pro-inflammatory foods, specifically sugars, increase systemic inflammation and directly cause micro-ischemic damage to small blood vessels. Consumption of these foods can accelerate the physiological process of ARHL in the elderly. Interestingly, they did not find a significant relationship between intake of anti-inflammatory foods such as vegetables, fruits, and nuts and smoking and BMI with ARHL, although ARHL patients had a lower intake of these foods. It has been suggested that limiting pro-inflammatory foods may be more effective than relying on the protective effects of anti-inflammatory or anti-oxidant foods in preventing age-related diseases (13). On the other hand, other studies have reported the effect of nonsteroidal anti-inflammatory drugs (NSAIDs) on ARHL. Research conducted on nearly 27,000 men (aged 40–74 years) over a 20-year period starting in 1986 revealed that frequent use of anti-inflammatory drugs such as NSAIDs might increase the risk of hearing loss and tinnitus, particularly in middle-aged men under the age of 60. It has been suggested that this is because NSAIDs can reduce cochlear blood flow, and this type of drug can also be ototoxic in high doses (15).

In addition to diet, anxiety, and inflammatory factors, other studies have examined the effects of other factors such as smoking and alcohol on hearing loss in the elderly. There are conflicting reports about the effect of smoking and alcohol on the progression of ARHL. For example, in a 2020 study, a higher intake of alcohol but not smoking has been found among participants with ARHL (13). However, in the study by Kim *et al.* (2020) on the elderly population, neither smoking nor alcohol were associated with a greater risk of hearing loss (16). In addition, cross-sectional and survey studies have suggested an association between ARHL with vitamin intake or status (17-24) and folate (24-27) with conflicting results. Some failed to find any association between folic acid (28) or vitamin B-12 intake (28, 29) with ARHL. There are also studies that suggest ARHL is less prevalent in women using calcium channel blockers (30), and in those using cholesterol-lowering medication like statins (31), omega (32), and postmenopausal women who do not use (33, 34) or use (35, 36) hormone replacement therapy. Also in people with higher serum aldosterone (37), higher plasma melatonin levels (18) and higher plasma very long-chain n-3 PUFAs (38), lower risk of ARHL has been reported. The association of many genes with the progression of ARHL has been also demonstrated in several studies (39, 40) that may be potential target genes for the treatment of ARHL. 

Based on these studies that have found a relationship between ARHL with several lifestyle factors, as well as inflammatory and genetic factors, numerous interventional studies have been conducted in recent years to establish prevention and treatment strategies for ARHL. Due to the fact that ARHL is a multifactorial disorder and that multiple cell-death pathways (e.g., oxidative stress, calcium signaling, glucocorticoid signaling, sex-specific hormones, and stress response signaling pathways) can contribute to cell death and thus ARHL (41), multiple types of research utilizing various methods for prevention and treatment of ARHL have been executed over the years. This review will comprehensively explain the interventions for ARHL with their potential mechanisms.

**Method**

A comprehensive narrative review was carried out. We searched the electronic databases of Elsevier, Medline, Embase, Scopus, Web of Knowledge, Google Scholar, Clinical Trials, and the Cochrane database of systematic reviews using the terms age-related hearing loss (ARHL/AHL), presbycusis, prevention, treatment, and therapy using the Boolean operators AND/OR for relevant articles. In addition, bibliographies of extracted papers were also extensively searched for other potentially relevant studies reporting any type of intervention for ARHL were identified and included in this paper. All related articles were selected and reviewed. This is a narrative review that included all animal and human studies related to interventions targeting slowing down or prevention or treatment of ARHL. There were no other criteria for inclusion and exclusion of studies.

## Results

**Figure1:**
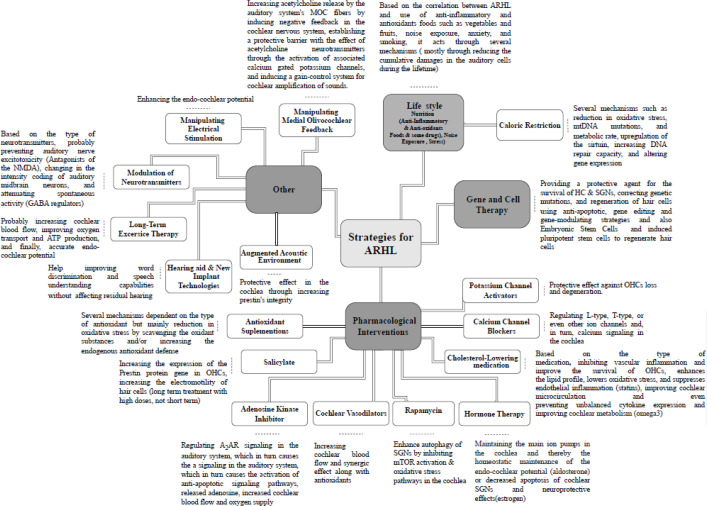
Interventions for age-related hearing loss with potential mechanisms

Numerous interventional studies have been conducted in recent years to establish prevention and treatment strategies for ARHL. These studies have primarily included caloric restriction (6, 42-53), anti-oxidant supplementations (5, 42, 54-78), gene therapy approaches targeting apoptosis and oxidative stress (79-97), and stem cells (98, 99). Although none of these methods have been implemented into clinical practice yet due to some limitations mentioned below, other strategies have also been evaluated in studies. However, these strategies are only at the theoretical level and were conducted in limited studies, some of which were designed based on cross-sectional studies that have found a relation between ARHL and some factors such as nutrition, inflammatory factors, taking some drugs, etc. These strategies include pharmacological interventions such as calcium channel blockers (100-102), statins (103), cochlear vasodilators (61), adenosine kinase inhibitors (104, 105), salicylate (106), rapamycin (107-110), and some other strategies such as hormone therapy (111-115), augmented acoustic environment (AAE)(116-118), modulation of neurotransmitters (e.g., GABA up-regulation) (119), Modulation of Metabotropic Glutamate Receptor 7 (120), electrical stimulation for restoring the endo-cochlear potential (121), long-term exercise therapy (122), enhancing medial olivocochlear feedback (123), and also new methods in amplification such as implant of vibrant sound-bridge (124). Multiple mechanisms have been postulated for each strategy. It should be noted that there are two often separate but clearly different terms, prevention versus treatment in research on aging and age-related disease. In ARHL, also both lines of research have been conducted. The detail of interventions will be discussed in the following sections as illustrated in Figure 1.

***Interventions for ARHL***

*Caloric restriction*

Among interventions, more studies have been conducted on caloric restriction and anti-oxidants. Caloric restriction, a long-term reduction in caloric intake, has been shown to delay the onset of age-related disease and extend the maximum lifespan (6, 43, 44). Several interventional studies have evaluated the effect of caloric restriction (CR) (6, 42-52). According to caloric restriction studies, Sirt3, a mitochondrial Sirtuin, increases NADPH production, primarily leads to enhancement in the mitochondrial glutathione anti-oxidant defense system, and could finally decrease oxidative stress-induced cell death and prevent ARHL. Interestingly, there are some signaling molecules like N1-methyl nicotinamide (MNAM) that could up-regulate SIRT1 and SIRT3 expression in the cochlea, and its related increased enzyme levels under caloric restriction conditions could also be a therapeutic candidate for ARHL. Recently, it has been found that supplementation with MNAM could delay ARHL via this exact mechanism of caloric restriction. They also showed that a high-fat diet accelerates ARHL (125). In contrast, Fujita *et al*. (2015) proved that, compared, to caloric restriction, a high-fat diet postpones ARHL progression in C57B/6J mice but not in CBA/N-slc mice. They hypothesized that a high fat intake contributes to vitamin E’s anti-oxidant effect in the inner ear, thereby delaying ARHL progression in C57BL/6J mice (126). However, the results of studies that suggest that caloric restriction delays aging and ARHL are numerous and this finding is more consistent with the cross-sectional studies.

*Anti-oxidants*

Based on some reports about lower prevalence of ARHL in vitamin users and the vital demonstrated role of oxidative stress in ARHL, several interventional studies have evaluated the effect of anti-oxidants (5, 42, 54-78) on ARHL. These studies were mostly based on cross-sectional and population-based studies that showed an association between ARHL and nutrition. These studies suggested a better diet consisting of fruits and vegetables containing many anti-oxidants that reduce the risk of ARHL. We reviewed the interventions with anti-oxidants in the prevention of ARHL described fully in our previous paper and found that the preventive effect of anti-oxidant supplementation against ARHL has been inconsistent across reports (127). This is probably because the effect of anti-oxidants differs depending on several factors such as the strain of mice (52, 126), rate, duration, the onset of treatment, and single or combination of used anti-oxidants(128). In addition, there are some limitations to anti-oxidant therapy. For example, for those who have reached their anti-oxidant capacity through optimal nutrition and adequate physical activity, anti-oxidant supplementation may provide no extra benefit, at least in terms of ARHL (7). However, studies on anti-oxidants are ongoing. A large number of natural plants that contain a high amount of anti-oxidants have also been evaluated for ARHL which include Silymarin (129), Ninjinyoeito (130), Ginko Biloba (71, 131), Red ginseng (132), Erlong Zuoci (133), oolong tea (134), plants or compounds containing polyphenols or flavonoids (65, 73, 77, 135, 136), Soybean (137), Thymoquinone (78), and Yi-qi Cong-ming (YQCM)(138) with sometimes conflicting results. Some are usually multifunctional agents and act through different mechanisms. In general, most of anti-oxidants can scavenge oxidant substances, thereby avoiding the potential damage associated with oxidative stress caused by aging in the auditory system. However, reducing oxidative stress and increasing the endogenous anti-oxidant defense is done using different ways including anti-oxidant-rich nutrition, pharmacotherapy, and gene therapy which will be discussed in the following section.

***Gene and cell therapy***

*Gene-therapy*

As mentioned, there are also other methods for enhancing the anti-oxidant capacity of the body via gene expression. Several novel strategies utilizing gene therapy to decrease apoptosis in auditory system disorders are now being developed. These methods target auditory system protection as well as hair cell regeneration. Gene therapy is performed for various purposes in ear diseases, such as providing a protective agent for the survival of hair cells and ganglion neuron cells following injury, correcting genetic mutations, and regeneration of hair cells.

ARHL and other non-syndromic sensorineural hearing loss disorders are directly associated with hair cell irreversible death. Several researchers have expressed interest in treating ARHL through the protection and regeneration of hair cells. Multiple possible gene therapy pathways have been identified due to extensive research on hair cell regeneration. Many studies have shown that mutations in some genes may increase susceptibility to ARHL and showed the effect of genetic predispositions for ARHL. Different techniques have been conducted to identify genes associated with ARHL and found possible association of many genes with the progression of ARHL such as DFNA5, MYO6, GRM7, GRHL2, KCNQ4, SLC26A4 (39), and CDH23 (40). Collectively, some potential candidate genes related to ARHL may be found in genes related to inner ear structures and/or inner ear cells (KCNQ4 gene encoding the voltage-gated K+ channel, SLC26A4 encoding an anion transmembrane transporter), some in genes related to inner ear oxidative stress (e.g., mtDNA4977) (39). These as well as other genes may be potential target genes for the treatment of ARHL by gene therapy, and some are used to model ARHL and new treatments. For example, a new mouse mutant of the CDH23 gene with early-onset hearing loss has emerged to facilitate the evaluation of otoprotection drugs on some form of inner ear disease linked to mutations in the *CDH23* gene such as ARHL (83). Down-regulating or up-regulating of related genes that can be used for intervention in ARHL using several gene therapy strategies have emerged that have shown promising therapeutic results for non-syndromic sensory-neural hearing loss, which mainly include viral and non-viral, gene editing, and gene-modulating strategies.

*Anti-apoptotic treatments*

There are also strategies for ARHL that target decreasing apoptosis in the cochlea to prevent age-related impairment. Because of the apoptotic program’s contribution to ARHL, over-expression of some apoptosis-related genes like Bak and Caspase-3 tend to be ARHL treatments. Anti-apoptotic treatments like caspase inhibitors and BCL2-antagonist/killer1 (Bak) inhibitors act by inhibiting apoptotic-related gene expression, and they may protect against progressive age-related degeneration in the cochlea (83-87).

Using Cdh23 mutant mice model, some studies found the otoprotective effects of some agents like erythropoietin (EPO) against ARHL (79, 84). EPO is a multifunctional cytokine hormone featuring anti-apoptotic and neuroprotective properties. They demonstrated that EPO might have otoprotective properties in Cdh23erl/erl mutant mice by assessing the ABR thresholds and amplitudes of distortion product otoacoustic emission (DPOAE) by preventing apoptosis in erythroid progenitor cells, its cytoprotective or otoprotective mechanism seems to be anti-apoptotic (84). Epo-transgenic mice had dramatically reduced spiral ganglion neurons compared with the control group, mainly in the basal turn. In addition, there were better preservative inner and outer hair cells in Epo-transgenic mice. Therefore, it has been suggested that EPO can effectively suppress the loss of spiral ganglion cells and probably hair cells and, thereby, the development of presbycusis in mice (79).

Similarly, it has been found that using a pan-caspase inhibitor such as Z-VAD-FMK and TUDCA in Cdh23 mutant mice model can effectively reduce apoptosis in the cochlear tissues. Z-VAD-FMK significantly reduced outer hair cell loss (83, 85, 86) and preserved hearing of more than 10 dB SPL (86) and about 15 dB SPL (85), and up to 35 dB SPL for the ABR thresholds (83). In a study by Hu *et al*.(2016), TUDCA significantly reduced hearing loss and inhibited hair cell death in CDH23 mutant mice cochlear, owing to its ability to block apoptotic genes and caspase-3 activation. The authors indicate that TUDCA treatment is equivalent to or perhaps superior to erythropoietin and Z-VAD-FMK treatment for hearing improvement (as determined by ABR threshold shift) (87). TUDCA is a taurine-conjugated bile acid that is derived from Ursodeoxycholic acid (UDCA). From many years ago, UDCA has been extracted from dried black bear gallbladders and administered in traditional Chinese medicine to treat various ailments. TUDCA was extensively administered in clinical and laboratory research to treat liver disease, diabetes, and neurological illnesses. TUDCA was previously shown to act by altering the apoptotic threshold in various cell types (87). Another study showed the role of another gene, Efr3a, in ARHL. Efr3a is involved in the degeneration of several structures. The findings showed that down-regulation of Efr3a improves auditory function and suppression of the degeneration of SGNs in an early stage of senescence, probably via blocking of cell apoptosis (81). 

A series of other interventions has focused on reducing oxidative stress and apoptosis *by* selectively decreasing or increasing the expression of specific genes or increasing the expression of anti-oxidants in the cochlea. A recent study showed redox activation in the auditory excitatory pathways, and NOX activity leads to excitotoxic cochlear damage and ultimately to ARHL. In this study, the down-regulation of NOX subunit p22^phox ^in auditory neurons conserved hearing and cochlear morphology (90). Thus, it may be a future therapeutic target for ARHL, as NADPH oxidases (NOX) are critical sources of reactive oxygen species (ROS) in the cochlea and may thus play a role in the pathogenesis of the disease (90). NADPH is predominantly synthesized by the enzyme G6PD (glucose-6-phosphate dehydrogenase). Another recent study revealed that G6PD might have a role in preventing ARHL. Anti-oxidant enzyme gene expression was dramatically increased in G6PD-Tg mice compared to wild-type controls. However, pro-apoptotic protein levels were suppressed in this group. During aging, G6PD-Tg mice exhibit lower hearing thresholds than wild-type mice, as well as preserved IHCs and OHCs, OHC innervation, and a conserved number of synapses per IHC. Protein nitration, mitochondrial damage, and apoptotic cells labeled with TUNEL were all much reduced in the G6PD-Tg mice group. They determined that overexpression of G6PD is related to cochlear cellular degeneration because it increases NADPH synthesis, which helps maintain a balance between free radical production and cellular detoxification throughout aging, hence reducing the development of hearing loss (91). 

Suppressing oxidative stress using a master transcription factor, NRF2 which regulates various anti-oxidant proteins has also been evaluated for ARHL, recently. The findings showed that elevation of NRF2 activity through KEAP1-inhibiting can protect the cochlea from oxidative damage during aging, particularly at the apical and middle turns (92). A study suggested that NRF2 can protect the inner ear against age-related hearing injuries and gentamicin ototoxicity by up-regulating anti-oxidant enzymes and detoxifying proteins (80).

Oxidative stress can induce mitochondrial dysfunction, cell death, or apoptosis. As a fundamental principle in the field of molecular cells, the research results show that proper metabolic and anabolic function in the cell, in which the mitochondria play a significant role, is one of the most critical factors in preventing the onset of ARHL and cell degeneration cycle. A variety of critical events on apoptosis focus on mitochondria. Studies have shown that the two mechanisms of mitophagy and mitochondrial biogenesis are the most crucial cell death mechanisms activated during oxidative stress and aging. These two mechanisms are in contrast and are directly controlled and modulated by MicroRNA-34 (miR-34a) and Sirtuin 1 (SIRT1). Suppose the amount of these two control factors stays accurate and in its perfect balance, in that case, it will cause a balance in the two mentioned mechanisms, which will prevent ARHL (93). Activation of Sirt1 expression was also one of the signaling pathways involved in the action mechanism of caloric restriction’s effect on ARHL. Sirt1 activation has been shown to activate the autophagy system, minimize hair cell death and hearing loss, and prevent the development of ARHL in animal models. In a 2019 study, pharmacological inhibition of miR-34a/SIRT1 signaling elevated the mechanism of mitophagy, and mitochondrial biogenesis and thus reduced cochlear cell death due to oxidative stress. In contrast, prolonged use of resveratrol, as a SIRT1 activator, caused a reduction in the rate of hair cell death during aging, spiral ganglion neuron loss, and stria vascularis atrophy, and prevented the increase of auditory thresholds during the aging process. In addition to the mechanisms mentioned above, overexpression in SIRT1 or miR-34a deficiency both reduced the death of hair cells due to aging and prevented hearing loss significantly (93). As mentioned before, other supplements, such as N1-methylnicotinamid (MNAM), can also activate cochlear SIRT1 expression and prevent ARHL(125).

Using heat-shake proteins is another way to inhibit apoptotic pathways by affecting mitochondria. This method has also been evaluated in ARHL using pharmacological up-regulation of HSPs. In a study by Mikuriya *et al*., increasing expression of HSP70 and HSP110 by heat shock protein inducers, geranylgeranylacetone, ameliorated ARHL in DBA/2J, which has been proven in ABR test and histological examination. However, the protection was specific to the apical portion of the cochlea (88). Hsp110, along with Hsp70, prevents proteins from breaking down in cells. Decreased gene expression levels and construction of hsp70 and hsp110 can cause hearing loss. Therefore, using pharmaceutical methods to increase these two will probably prevent the aging process effectively (88). Recently, a study also found that HSF1 can function as a mediator to prevent ARHL by decreasing ER stress-dependent apoptosis in the aging cochlea (89). The research on heat-shake proteins needs further studies. 

In addition, there are numerous genes responsible for coding specific supporting factors such as Brain-Derived Neurotrophic Factor (BDNF) and Neurotrophin 3 (NT-3), or Glia-cell Line Derived Neurotrophic Factor (GDNF) that are involved in the survival rate of Spiral Ganglion Neurons (SGNs). In a recent study, it has been found that increasing cochlear NT-3 levels in the middle-aged ear can prevent the age-related degradation of auditory responses, maintaining IHC and SGNs synapses and their function(96). 

*Cell therapy*

The other approaches in treating sensory-neural hearing loss focus on hair cell regeneration. Many attempts have been conducted to induce hair cell regeneration in adult mammals using over-expression of specific transcription factors such as Atoh1 (139, 140). In fact, over-expression of the Atoh1 gene in the inner ear via genetic or pharmacological manipulation has been shown to induce the trans-differentiation of the supporting cells in the cochlea to new hair cell formation and restore hearing in experimental animal models (140). Many types of research have focused on Atoh1 for regenerating hair cells and improving this method as one option for sensory neural hearing loss, including ARHL. However, the findings are controversial. Recent studies also suggest that manipulating other transcription factors can increase efficacy compared with Atoh 1 alone. A study by Walters *et al*. (2017) showed that manipulating other factors such as p27^Kip1^, GATA3, and POU4F3, in addition to Atoh 1, can be more effective in trans-differentiation of the supporting cells to new hair cells and treating ARHL and NIHL (141).

One of the other hair cell regeneration methods is using stem cells. Many studies have evaluated the treatment of inner ear disease using stem cells from less common methods such as Embryonic Stem Cells (ESC) and induced pluripotent stem cells (99) to newer and more efficient methods such as Mesenchymal Stem Cells (MSC) (142). Stem cells are the foundation basis of all organs and tissues in the body. Different types of stem cells are divided according to where they are extracted and what they can become. However, in ARHL especially, limited studies have been conducted. An embryonic neural stem cells transplantation (NSCs) study demonstrated improvement in the auditory of C57BL/6J mice with presbycusis and a lesser neuronal rate of apoptosis in NSCs transplantation (98). Another study integrated the *Cdh23* gene containing a uniquely targeted nucleotide with embryonic stem (ES) cells and evaluated the effect on ARHL. They found the role of the *Cdh23*^c.753G^ allele in preventing the progression of high-frequency hearing loss in only one strain type but not all strains (99).

Gene therapy and stem cells are definitely some of the promising future approaches. They can inevitably be ideal solutions for the prevention and treatment of gene-related diseases. Still, it should not be overlooked that gene therapy is associated with many challenges and risks that can have the opposite effect on the treatment process. One of the challenges is safety considerations about potential side effects. Vectors are being used in the gene therapy process to facilitate gene transfer; these vectors, in turn, can cause various effects on non-target cells. So all gene and cell therapy methods must be adequately controlled to avoid destructive cell growth. Another issue is the high cost of these therapies, which is a fundamental challenge in gene therapy: the general public will not be able to afford the high price. Finally, the effectiveness of the methods is still questionable. Despite all the challenges, gene and stem cell therapies are promising strategies for treating sensory-neural hearing loss because they are the only options to regenerate hair cells in the mature mammalian cochlea.

The rest of the strategies are only at the theoretical level due to the limited studies. 

***Pharmacological interventions***

*Calcium canal blockers*

ARHL has been reported less prevalent in women using calcium channel blockers. In according, interventional studies provided further evidence that T-type calcium channel blockers could effectively prevent ARHL (100-102). In a study by Yu *et al*.(2016), administering a T-type calcium channel blocker for four weeks caused a significant improvement in DPOAE amplitudes and 24 kHz  ABR thresholds and improvement in the function and morphology of the OHCs but not IHCs (101). The underlying mechanism is the vital role of calcium signaling in the auditory system. Calcium canal blockers may affect L-type, T-type, or even other ion channels in the cochlea (101). They have been shown to protect neurons using ameliorating calcium overload on spiral ganglion neurons (100).

*Potassium channel activators*

The function of the auditory system and hair cells is highly dependent on different channels. One of these channels is the Kv7.4 (a voltage-gated potassium channel) channel, which expresses in OHCs. A recent study showed that pharmacological activation of this potassium channel using ACOU085 (a novel small-molecule agonist of the K_V_7.4) can significantly reduce age-related threshold shift of auditory brainstem responses as well as OHC loss in a SAMP8 mouse model. Targeting the important channels in the organ of corti can be a potential therapeutic option for ARHL. Further studies are needed in this regard (143). The mechanism was attributed to the protective effect of the agonist of the K_V_7.4 against OHCs loss and degeneration. This is because the survival of OHCs is dependent on the functional circuit of potassium.

*Cholesterol-lowering medication*

In literature, a relationship between cholesterol-lowering medications like statins and omega and the risk of ARHL has been reported (31, 32). It has been suggested that statins can inhibit vascular inflammation, slow ARHL, improve the survival of outer hair cells, and achieve larger amplitudes of DPOAEs (31). 

So far, the effect of long-term omega supplementation has been researched, and it has been proven to preserve the cochlea and prevent hearing loss. In a study omega fatty acids prevented unbalanced cytokine expression toward pro-inflammatory cytokines during cochlear aging and may also partially prevent the progression of hearing loss. Compared to the control group, the omega-3 group had significantly lower ABR hearing thresholds and significantly greater DPOAE amplitudes in the mid to high frequencies (144).

Honkura *et al.* also established considerable evidence for the protective effect of enhanced endogenous n-3 PUFAs against ARHL. They concluded that enhanced endogenous n-3 PUFAs created as a result of Fat-1 transgenic expression may be beneficial in preventing ARHL in male C57BL/6N mice(145). In studying the effect of nutrition and diet on hearing health, the role of maternal nutrition during pregnancy and after pregnancy cannot be ignored. Research has shown that Omega-3 fatty acid over-nutrition or imbalance has adverse neurological and sensory function effects during adulthood and aging (146). The effect is probably related to omega’s effect on cochlear metabolism (144).

In a review study by Tang *et al.* (2019), nutritional interventions for obesity and comorbidities, including a low-fat diet, statin supplementation, omega-3 polyunsaturated fatty acids, and alpha-lipoic acids, were reviewed to explore the protective effect of nutritional interventions for obesity against the development of ARHL. They discovered that a high-fat diet may trigger oxidative stress, mitochondrial damage, and inner ear apoptosis. Statins, on the other hand, have been proven to slow the progression of ARHI by enhancing the lipid profile, lowering oxidative stress, and suppressing endothelial inflammation. By reducing dyslipidemia and preventing inflammation, omega-3 polyunsaturated fatty acids may help protect the cochlear microcirculation (147). 

*Cochlear vasodilators*

The presence of ion balance and blood flow in the human cochlea is vital. Studies have shown a reduction in cochlear blood flow and vascular conductance during aging, and some also suggest that alterations in the stria vascularis could be the primary cause of hearing loss during aging (61). Vasodilators could regulate cochlear blood flow. It has been suggested that both medicinal and organic vasodilators, such as magnesium, can significantly restore blood flow to stria vascularis, restoring the endocochlear potential to its equilibrium, stabilizing ionic balances, and thereby improving sound amplification. Researchers suggest that using cochlear vasodilators with natural anti-oxidants together could have synergic effects and provide promising effective therapeutic options for ARHL (61). Due to the weakness and various disorders in the circulatory system of the elderly and the delicate structure of the human cochlear blood supply network, the importance of blood flow and the use of cochlear vasodilators in the prevention of ARHL has become much clearer but needs further studies.

*Salicylate*

Inflammation in the human immune system is a natural barrier against various diseases, infections, and tissue damage. This factor is now mentioned as one of the most critical factors in many diseases and physiological disorders, including noise- and drug-induced ARHL. According to studies, improving the resolution phase of the inflammatory response in the inner ear can lead to cell protection at the cochlea and prevent and even treat hearing loss caused by the aging process (148). Recent studies have discovered associations between hearing loss and inflammatory markers such as C-reactive protein, IL-6, and TNF-α, all of which indicate an inflammatory state in human case-cohort studies. It has been suggested that one of the most accessible and effective ways of treating ARHL is to improve the resolution of cochlear inflammatory responses. The limitations and complexities of inflammatory processes in the face of invasive organisms and the repair of damaged tissues all lead us to manipulate and regulate the inflammatory process. According to studies, improving the resolution phase of the inflammatory response in the inner ear can lead to cell protection at the cochlea and prevent and even treat hearing loss caused by the aging process. The aging process’s anatomical and physiological effects on the ear’s physiology and a better understanding of inflammatory processes may suggest newer and more effective solutions in the future field of treatment and prevention of ARHL (148). Recently, the effect of leukocytes and immune cells such as CD4^+^ T cells (*an* essential mediator of immune homeostasis and *inflammation)* on ARHL has also been investigated. It has been found that injections of a lymphoid fraction can prevent ARHL. It can suppress cochlear degeneration and serum nitric oxide levels (149). 

Conflicting results have been found regarding the use of anti-inflammatory drugs and ARHL. Significantly, the effect of NSAIDs like salicylate has been evaluated on the auditory system. Salicylate protects against ototoxicity and noise-induced hearing loss (106). It has been also found that salicylate induces cochlear temporary sensitivity loss and a reversible OHC dysfunction after acute administration in aged rats. However, long-term treatment with high doses of salicylate might exert different effects on the auditory system than acute treatments (106). Prolonged treatment with high doses of salicylate increases the expression of the prestin protein gene in OHCs, increases the electromotility of hair cells, and thus enhances DPOAE with a permanent amplitude reduction in CAP (compound action potential) and ABR responses. A fascinating finding from the high-dose treatment of salicylate in mice was that if the interval between doses were increased, the reducing effects on ABR and CAP in mice would be eliminated, and the positive effect on DPOAE would remain, resulting in increased sensitivity of the auditory system (106). The research results on salicylate effects are still vague and unknown in many cases and need to be examined in more detail. In addition, the effect of regulating the immune response to prevent ARHL needs further studies. 

*Rapamycin*

Rapamycin is an mTOR (Mammalian Target of Rapamycin) inhibitor distinguished mainly through its immunosuppressive properties. It operates on additional functional signaling pathways, including those linked to metabolism, proliferation, immunological response, and cell survival. It has been proposed as a potential treatment option for age-related diseases. Altschuler *et al*. (2018, 2021) showed that a later-in-life injection of Rapamycin could delay age-related loss of outer hair cells and ARHL in mouse models by modulating the mTOR signaling pathway, a crucial determinant in the development of ARHL. However, they claimed this reduction is more of a delay/deceleration than full prevention. Rapamycin has a “delay” rather than a “prevent” effect on ARHL. Rapamycin could also boost the survival pathway of p-Akt (S473) via mTOR2 signaling, blocking oxidative stress pathways in the cochlea and acting via its influence on inflammation (107, 108). However, Rapamycin’s adverse effects, such as immunosuppression, pose an essential dilemma regarding its use (109). Rapamycin also promotes autophagy of SGNs by suppressing mTOR activation, resulting in improvement in ARHL (110). 

*Adenosine inhibitors:*

Adenosine signaling is known to decline in the aging brain, and a similar process has been postulated to occur in the aging cochlea (104, 105). Currently, almost all new and innovative ARHL research is being conducted on animal species, specifically the C57BL / 6 mouse model. This mouse model is precisely suitable for designing and simulating ARHL in animal models. A recent paper discusses the effects of Istradefylline as an adenosine inhibitor on the prevention and treatment of ARHL (105). Istradefylline is an FDA-approved drug widely used in clinics to treat the symptoms of Parkinson’s. They investigated the effects of the A2AR antagonist Istradefylline on ARHL in C57BL/6 mice and, in parallel, placed cognitive function in the Istradefylline-treated mice group to exclude the neurotoxic effects of the treatment. After six months of weekly use of this drug, the results of the effect of this drug in four areas of body weight, ABR responses, hair cell survival, and behavioral responses were evaluated. ABR responses indicated significantly lower threshold shifts at high (32 kHz) and middle frequencies (16 kHz) in Istradefylline-treated mice compared to the control group. Response alterations were also found at low frequencies but were not statistically significant. Hair cell numbers decreased much greater in outer hair cells than those in inner hair cells in both groups. However, in the Istradefylline-treated group, this drop in the number of hair cells in the middle and basal turns was substantially less than in the control group. However, there was no change in the number of apical cells (105). Additionally, another study established the significance of adenosine signaling in ARHL. The study demonstrated increased adenosine signaling in the cochlea, which resulted in partial alleviation of ARHL in C57BL/6J mice treated with ABR, as well as increased hair cell survival in the apical region of the cochlea (104).

The mechanism enhanced by this drug might be A_2_AR inhibition. A_2_AR signaling and its effects on the brain and ears are still not fully understood and discussed, but its effects have been confirmed in many cochlear physiological functions such as blood flow and sound conduction. Researchers suggest the underlying mechanism action of adenosine inhibition in the cochlea is activation of anti-apoptotic signaling pathways, released adenosine,  increased cochlear blood flow and oxygen supply via A_2A_ receptors, and enhanced anti-oxidant defenses via A_1_ receptors that in turn improve the cochlear function during the aging process (104).

***Other strategies***

*Hormone therapy*

Hormones have been studied earlier in relation to auditory function, and it has been claimed that hormonal levels may impact ARHL. As a result, hormone therapy has been suggested as a possible treatment option for ARHL. In agreement with some cross-sectional or cohort studies that showed ARHL is less frequent in those with higher serum aldosterone levels and postmenopausal women who receive hormone replacement therapy, some experimental studies have demonstrated that aldosterone hormone therapy is helpful for cochlear function in aging (111, 112). Frisina *et al*. demonstrated that aldosterone has therapeutic effects on spiral ganglion neurons by increasing their survival, up-regulating mineralocorticoid receptors, and finally, inhibiting apoptosis in the cochlea of mice (111).  It has been suggested that it may delay or reverse the metabolic pathogenesis of the stria caused by aging by maintaining the main ion pumps in the cochlear lateral wall cells, including NKCC1 (Na-K-Cl co-transporter 1), which is involved in the homeostatic maintenance of the endo-cochlear potential. This is because aldosterone regulates the expression of the sodium (Na+) and potassium (K+) ion transporters NKCC1 and Na-K-ATPase and thereby the level of sodium and potassium (111, 112). Researchers evaluated the influence of estradiol and progesterone hormones on middle-aged female CBA/CaJ mice (113). They discovered that animals treated with estradiol had negligible ABR threshold changes in both HRT and recovery period compared with the placebo and all of the other HRT groups. Additionally, both groups of animals treated with estradiol and progesterone had elevated insulin growth factor 1 receptor (IGF1R) expression in the stria vascularis. They determined that hormones, particularly estradiol, have neuroprotective qualities in the auditory system that may help prevent or delay certain types of presbycusis. However, progesterone has no determining effect on the auditory system. The underlying process is a shift in the rate of IGF1R expression in the mammalian cochlea, which could increase sensory cell health and delay certain crucial features of ARHL via the PI3K/AKT pathway (113). Another study discovered that a four-month combination of estrogen and progestin hormone treatment impairs the function of outer hair cells and overall auditory sensitivity, potentially accelerating ARHL, compared to estrogen monotherapy (114). A recent study also revealed that estrogen treatment might protect against ARHL as estrogen-treated aged C57BL/6J mice demonstrated decreased apoptosis of cochlear spiral ganglion cells and hearing threshold (115).

*Neurotransmitters modulation*

Aging could lead to changes in levels of neurotransmitters (119, 120), so it seems modification of neurotransmitters could help to slow down the ARHL progression. Therefore, modulation of Metabotropic Glutamate Receptor 7 (120) and GABA up-regulations (119) have been suggested as innovational options to improve auditory function in ARHL. For example, L-glutamate is the major excitatory amino acid neurotransmitter in the mammalian central nervous system. Antagonists of the NMDA (iGluR) receptor, such as Dizocilpine, have been shown to prevent auditory nerve excitotoxicity caused by noise, presbycusis, or aminoglycoside antibiotics (120). Another study showed that boosting GABA levels with agents such as Vigabatrin (VGB) restored age-related changes in the intensity coding of auditory midbrain neurons in elderly mice, attenuated spontaneous activity, and enhanced minimum thresholds while preserving frequency selectivity (119).

*Electrical stimulation to restore the endo-cochlear potential*

It has been claimed that by enhancing the e*ndocochlear* potential with DC voltage, hearing sensitivity in aged Mongolian gerbils can be recovered to approximately 40 dB(121). No other study has been found in this regard.

*AAE (Augmented acoustic environment)*

In 2009, Tanaka and colleagues published an article on the impact of acoustically controlled and enlightened environments on ARHL. They arranged two groups of Fischer 344/NHsd rats. The first group was exposed to 80 dB SPL stimulation for 12 hr a day and five days a week in an environment with 4 to 20 kHz frequency band sounds. The second group was kept in normal controlled daily conditions. Then, after 13 weeks of exposure to AAE, ABR tests were taken from them, and also the morphology of hair cells of the two groups was examined using propidium iodide and antiPrestin antibody staining. The results were interesting: in the group treated with AAE, the hearing thresholds in the 4 to 20,000 Hz range were less affected (1-3 dB) by ARHL than the control group. In terms of cell number and appearance, observations of hair cells in both groups were the same. Examination of prestins in these two groups resulted in an astonishing result; prestins density was more integrated and numbered than the control group in the AAE-treated group. Results highlighted the point that AAE increases prestin’s integrity and thus causes a protective mechanism against ARHL (116); further studies are needed.

*Long-term exercise and physical activities*

As a major part of aging, exercise and physical activity have consistently shown significant and proven results in various fields of research. The nature of these sports activities is tied to their direct and undeniable impact on the musculoskeletal system, circulatory system, and body homeostasis systems in preventing various diseases related to aging disorders and non-aging ones. It is true that so far, no long-term human-phase research has been conducted on this issue. However, animal research confirms this two-way relationship between ARHL and physical activity (122).

In an experiment researchers exposed a group of mice from 6 months to 24 months of age to an 18-month voluntary wheel running. Their level of sports activity at 24 months was calculated at about 3987 m/d. After comparing the WR group and the control group, the results, as expected, showed that the middle and lower levels of hearing loss were lower in the ABR thresholds of the WR group of aged mice. Additionally, laboratory data revealed that the loss of cochlear hair cells and spiral ganglion neurons was significantly less likely to occur in the exercise group than in the control group. Gene ontology observations also showed quite clear enrichment at the level of the immune response, inflammatory response, vascular function, and apoptosis-associated genes. In confirmation of the results obtained from gene ontology, the number of oxygen-carrying capillaries and cochlear nutrient factors and the severity of Stria Vascularis atrophy were quite evident in the control group compared to the Wheel Running group (122).

*Enhanced medial olivocochlear feedback*

The effect of aging on hearing can be explored in many ways. These effects comprise both neural and non-neural. Over time, the aging process affects the hearing perception of the older adult and dramatically reduces the person’s discrimination of sounds and speech stimuli in crowded and noisy environments. Cochlear denervation or synaptopathy is one of the causes of hearing problems mentioned in the discussion of ARHL. A recent article by Boero *et al*. discusses this type of neural ARHL. In the study conducted on several mice diagnosed with age-related cochlear synaptic degeneration and hair cell loss in mice, they were treated by enhanced α9α10 cholinergic nicotinic receptors gating kinetics. This method has an impressive effect on medial olivocochlear feedback by increasing acetylcholine release by the auditory system’s MOC fibers by genetically α9 nAChR point mutation. Medial olivocochlear feedback is a system that naturally induces negative feedback in the cochlear nervous system and establishes a protective barrier with the effect of acetylcholine neurotransmitters through the activation of associated calcium-gated potassium channels, then; due to this mechanism, the cochlea becomes sensitive to the auditory stimuli and acts as a gain-control system for cochlear amplification of sounds. The findings showed both DPOAEs and ABRs thresholds were improved in α9 knockin [α9KI] mice, and hair cell death and synaptic loss were significantly decreased in comparison to wild-type mice. The results also show the direct and positive effect of efferent feedback in maintaining and long-term stability of this system on the inner ear and the application of medial olivocochlear system enhancement as an effective method in preventing ARHL (123). Further studies are needed in this regard.

*Hearing aid and implants*

Apart from all the medical, genetic, and physiological methods, we cannot ignore the unique role technology plays in hearing, especially hearing aids and wide implants used in hard-of-hearing patients. The growth rate of technology has increased very much, and at the same time, this growth of technology in the technologies used in the field of rehabilitation and therapy in audiology has also increased very fast. The use of hearing aids is a ubiquitous method in the rehabilitation of hearing loss due to age. However, amplification with a hearing aid may not improve word discrimination and speech understanding capabilities in older adults with more severe hearing loss. As a result, implants may give an alternate approach to hearing rehabilitation. The number of cochlear implant users is increasing. The studies show that consistent use of cochlear implantation in adults causes an improvement in speech understanding abilities (150).

Although these approaches have improved communicative abilities, such as speech recognition abilities, not hearing improvement, a study by Haar *et al*. on aged dogs used another type of implant, the Vibrant Sound Bridge Middle Ear Implant, and showed improvement in ASSR thresholds. In this experiment, three old dogs with a mean age of 11.1 years were unilaterally implanted with VSB middle ear implants. After three months of implantation, the ASSR results showed 20.7, 13, and 16.3 dB improvement at frequencies 1, 2, and 4 kHz. In the inactive state of these implants, using brainstem-evoked response audiometry (BERA), the implants had no adverse effect on the residual hearing of the dogs. The audio processor used in these implants may not be strong enough for humans, but with a more powerful processor, these implants can be more efficient in the human phase and clinical use (124). Further studies are needed on the effect of the Vibrant Sound Bridge on ARHL.

***Conclusion and future directions***

**Figure 2:**
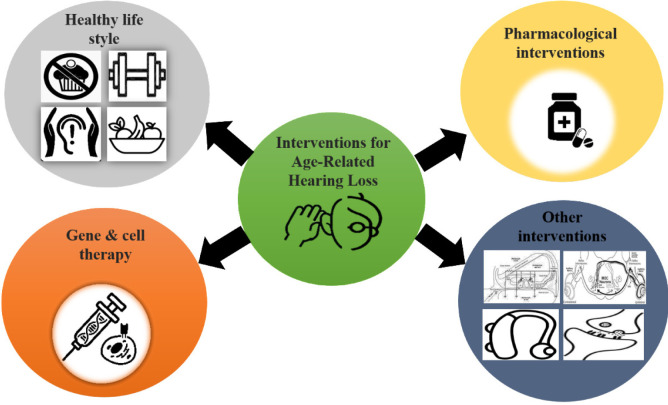
Summary of interventions for age-related hearing loss

Numerous methods have been studied for the treatment and prevention of ARHL, the results of which are sometimes positive and sometimes contradictory. Summary of interventions for ARHL was shown in Figure 2. Some studies have rarely been conducted, and most studies have been done on animals. Because several different underlying mechanistic pathways are associated with presbycusis which eventually leads to different types of this disease, a combination of different methods will probably be required and targeting only one pathway may be helpful. However, according to studies, it does not seem enough. It is unclear which approach/approaches are the best to slow down these dysfunctions. Because ARHL highly depends on intrinsic factors like genetics, the effectiveness of even some effective treatments may not have a considerable effect on some people or even be contraindicated. So, the effectiveness of some treatments for some people may need to be monitored. Therefore, we may need a unique and *personalized* approach to the prevention and treatment of ARHL for each person. In addition, each method should specify what type of presbycusis it affects, to what extent, the duration of treatment, its persistence, the side effects, and whether the approach is for treatment, prevention, or even both.

Currently, based on studies, it seems rational to control the modifiable factors related to ARHL, such as changing the lifestyle towards a healthy life by modifying unhealthy eating habits (e.g., reducing daily caloric intake and inflammatory foods as well as increasing anti-oxidant and anti-inflammatory foods**)**, minimizing noise exposures and anxiety during the lifetime to slow down ARHL. Although this may not guarantee complete prevention, the various treatment options developed so far need to be carefully monitored in humans. The treatment options of ARHL provided so far, either using novel pharmacological methods or gene therapy and stem cells, require further studies to explore the best way/ways of treatment with the most remarkable efficacy and the more negligible side effect in humans with the determination of details about the treatment procedure. The following concerns need to be addressed in studies in order to obtain the desired outcomes in the prevention and treatment of ARHL: what combination of medicines for what individuals, with what onset and duration, and with how much bearable side effects can be used? 

It should be noted that there are two often separate but clearly different terms, prevention versus treatment in research on aging and age-related diseases. In ARHL, also both lines of research have been conducted. Nevertheless, the efficacy of these strategies for ARHL is presently controversial and inconclusive, and further studies are needed.

## Authors’ Contributions

E T, V R, ME KH, S F, and GH M participated in all aspects of the main file draft. M MZ and E T participated in the revision of the final format of the article.

## Funding Sources

This work received no funding.

## Ethical Approval

In this review, all procedures performed in studies involving human participants or animals were in accordance with the ethical standards of the institutional and/or national research committee.

## Conflicts of Interest

The authors have no conflicts of interest to declare.
